# Heartfelt intervention: A role for PVN oxytocin neurons in the prediabetic heart

**DOI:** 10.1113/JP289973

**Published:** 2025-09-27

**Authors:** Grace K. Nielsen, Reesa M. Wilcox, Han‐Jun Wang

**Affiliations:** ^1^ Department of Anesthesiology University of Nebraska Medical Center Omaha Nebraska USA

**Keywords:** autonomic nervous system, hypothalamus, MASLD, oxytocin

Prediabetes affects an estimated 97.6 million adults in the USA, indicating that over one in three individuals are affected by an intermediate stage of glucose dysregulation. Both prediabetes and metabolic dysfunction‐associated steatotic liver disease (MASLD), driven by overnutrition from diets consisting of high fats and sugars, significantly increase the lifetime risk of developing type 2 diabetes (T2D) (Younossi et al., 2019). Altered insulin action, oxidative stress, and inflammation, stemming from physiological maladaptation to poor nutrition, lead to cardiovascular complications that occur in prediabetes and MASLD. Notably, prediabetic cardiomyopathy encompasses diastolic dysfunction, hypertension, endothelial dysfunction, and cardiac autonomic neuropathy that leaves the heart susceptible to future advanced cardiovascular disease. However, there is a lack of cardiac‐specific interventions, despite clinical trials emphasizing that cardiovascular health may not be restored to baseline after normalization of glycaemia (Kenny and Abel, 2019).

Recent seminal work by the team of Schunke, Kay and Mendelowitz demonstrated that targeting paraventricular nucleus (PVN) oxytocinergic neurons through chemogenic induction to manipulate cardiac cholinergic activation confers cardioprotective benefits in myocardial infarction (MI), hypertensive and obstructive sleep apnoea (OSA) models. The PVN of the hypothalamus is involved in regulating physiological processes by integrating interoceptive and exteroceptive signals through distinct subpopulations of neurons (Iremonger and Power, 2025). Both magnocellular and parvocellular neurons produce the neuropeptide oxytocin (OXT). Parvocellular pre‐autonomic neurons are involved in control of the sympathetic and parasympathetic nervous system via innervation of the rostral ventrolateral medulla (RVLM) and the dorsal motor nucleus of the vagus (DMNX), respectively. Cardiac vagal neurons (CVNs) are a main terminus of parvocellular pre‐autonomic neurons extending from the PVN. In rats, CVNs in the DMNX are activated by PVN neurons that co‐release OXT and glutamate which, in turn, excite OXT receptors on the CVNs, allowing PVN input to bypass sympathetic pathways and predominately stimulate parasympathetic tone.

In the current paper, Nilsson et al. (2026) further discovered that parasympathetic activity to the heart was found to be facilitated by these OXT‐expressing neurons in the PVN which synapse onto cardiac vagal neurons in the brainstem. Using male rats, a prediabetic model was used to evaluate the efficacy of PVN OXT activation on cardiac autonomic dysfunction. Administration of a high‐fat, high fructose (HFHF) diet induced metabolic and molecular effects to reflect the clinical parameters of prediabetes and MASLD, while lacking total disease progression to T2D. Excitatory designer receptors exclusively activated by designer drugs (DREADDs) were chronically activated with clozapine N‐oxide (CNO) to generate parasympathetic stimulation to the heart via cardiac vagal neurons – providing spatial and time‐dependent control to precisely target vagal neural activity (Nilsson et al., 2026).

The assessment of cardiac parasympathetic tone revealed that heart rate variability and resting heart rate, altered in the prediabetic condition, were restored to control levels by treatment. In contrast, blood pressure and exercise tolerance were unaffected by the development of prediabetes and therefore remained unchanged following treatment. PVN OXT neuronal activation through DREADD viral expression attenuated cardiac diastolic dysfunction and interstitial fibrosis of the left ventricle in the treatment cohort compared to disease. This neuronal activation did not affect the pathophysiology of the liver, nor the morphological or molecular changes in the pancreas.

DREADD‐mediated manipulation of the PVN OXT pathway revealed that cardiovascular autonomic function can be improved independently of metabolic status. This is compelling given the current therapeutic modality, vagal nerve stimulation (VNS), which has been tested in drug‐resistant epilepsy, depression, Crohn's disease and heart failure. The anti‐inflammatory and anti‐apoptotic effects of VNS may help limit cardiac fibrosis through preserved autonomic tone. However, direct VNS lacks cardiac specificity and risks off‐target effects.

PVN OXT neuron activation, by enhancing parasympathetic signalling pathways in the heart, demonstrates cardiac‐specific molecular and functional modifications. Intervention aimed at potential reversal of cardiac autonomic dysfunction in prediabetes and MASLD would greatly mitigate the risk of long‐term cardiovascular mortality in conjunction with, or even before weight management is controlled. Future research directions may be tailored to simulate the effects shown here by activation of the PVN OXT neuronal network with drug delivery through alternative methods (Fig. 1). This group previously explored intranasal delivery of OXT in OSA patients where it was found to decrease the severity and duration of obstructive events. Recent clinical trials have utilized intranasal delivery of OXT for weight loss (McCormack et al., 2023). While this trial produced mixed results, the medication was well‐tolerated in individuals and could provide a promising avenue for strategies that target cardiac autonomic dysfunction in metabolic disorders.

**Figure 1:**
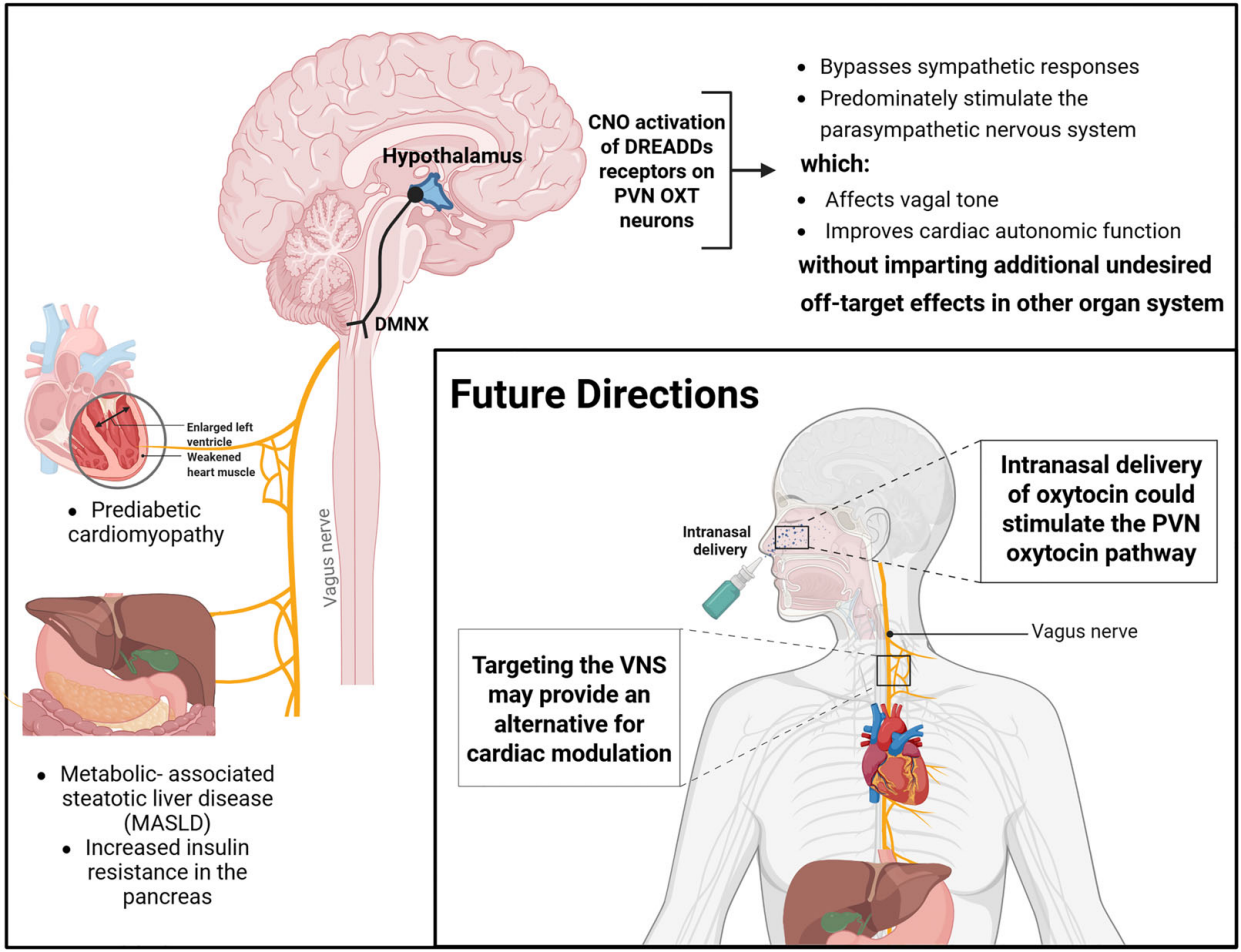
Neuroendocrine modulation in prediabetic cardiomyopathy A schematic depiction summarizing emerging strategies targeting PVN OXT neuron activation to treat prediabetic cardiomyopathy. Created in Biorender.

## Additional information

### Competing interests

The authors declare no conflict of interest.

### Author contributions

G.N.: Conception or design of the work; Drafting the work or revising it critically for important intellectual content; Final approval of the version to be published; Agreement to be accountable for all aspects of the work. R.W.: Conception or design of the work; Drafting the work or revising it critically for important intellectual content; Final approval of the version to be published; Agreement to be accountable for all aspects of the work. H.‐J. W.: Conception or design of the work; Drafting the work or revising it critically for important intellectual content; Final approval of the version to be published; Agreement to be accountable for all aspects of the work.

### Funding

HHS | NIH | National Heart, Lung, and Blood Institute (NHLBI): Han‐Jun Wang, R01 HL126796; HHS | NIH | National Heart, Lung, and Blood Institute (NHLBI): Han‐Jun Wang, R01HL169205; HHS | NIH | National Heart, Lung, and Blood Institute (NHLBI): Han‐Jun Wang, R01 HL172029; HHS | NIH | National Heart, Lung, and Blood Institute (NHLBI): Han‐Jun Wang, R01 HL171602; HHS | NIH | National Heart, Lung, and Blood Institute (NHLBI): Han‐Jun Wang, R01 HL152160; HHS | NIH | National Heart, Lung, and Blood Institute (NHLBI): Han‐Jun Wang, R21 HL170127 Funding This study was supported by NIH grants R01 HL126796, R01HL169205, R01 HL172029, R01 HL171602, R01 HL152160 and R21 HL170127.

## Supporting information


Peer Review History
