# Supplementary material for: Heartfelt intervention: A role for PVN oxytocin neurons in the prediabetic heart
Source: J Physiol. 2025 Sep 27;604(16):6565–6. doi: 10.1113/JP289973 (PMC13142777; doi:10.1113/JP289973)
Supplement: Supplementary file 1 [file TJP-604-6565-s001.pdf]

The Journal of Physiology

Heartfelt Intervention: A Role for PVN Oxytocin Neurons in the Prediabetic Heart

Grace K. Nielsen, Reesa M. Wilcox, and Han-Jun Wang
DOI: 10.1113/JP289973

Corresponding author(s): Han-Jun Wang (hanjunwang@unmc.edu)

The following individual(s) involved in review of this submission have agreed to reveal their identity: Kathryn J. Schunke (Referee #1)

**Review Timeline:**		
	Submission Date:	03-Sep-2025
	Editorial Decision:	08-Sep-2025
	Revision Received:	08-Sep-2025
	Accepted:	11-Sep-2025

Senior Editor: Bjorn Knollmann

Reviewing Editor: Beth Habecker

Transaction Report:

(Note: With the exception of the correction of typographical or spelling errors that could be a source of ambiguity, letters and reports are not edited. Depending on transfer agreements, referee reports obtained elsewhere may or may not be included in this compilation. Referee reports are anonymous unless the Referee chooses to sign their reports.)

1st Editorial Decision **08-Sep-2025**

Dear Dr Wang,

Re: JP-TP-2025-289973 "**Heartfelt Intervention: A Role for PVN Oxytocin Neurons in the Prediabetic Heart**" by Grace K. Nielsen, Reesa M. Wilcox, and Han-Jun Wang

Thank you for submitting your manuscript to The Journal of Physiology. It has been assessed by a Reviewing Editor and by 1 expert referee and we are pleased to tell you that it is acceptable for publication following minor revision.

Please advise your co-authors of this decision as soon as possible.

The review comments are copied at the end of this email.

Please address all the points raised and incorporate all requested revisions or explain in your Response to Referees why a change has not been made. We hope you will find the comments helpful and that you will be able to return your revised manuscript within 2 weeks. If you require longer than this, please contact journal staff: jp@physoc.org.

Your revised manuscript should be submitted online using the link in your Author Tasks: Link Not Available. This link is accessible via your account as Corresponding Author; it is not available to your co-authors. If this presents a problem, please contact journal staff (jp@physoc.org). Image files from the previous version are retained on the system. Please ensure you replace or remove any files that are being revised.

If you do not wish to submit a revised version of your manuscript, you must inform our journal staff (jp@physoc.org) or reply to this email to request withdrawal. Please note that a manuscript must be formally withdrawn from the peer review process at one journal before it may be submitted to another journal.

TRANSPARENT PEER REVIEW POLICY: To improve the transparency of its peer review process, The Journal of Physiology publishes online as supporting information the peer review history of all articles accepted for publication. Readers will have access to decision letters, including Editors' comments and referee reports, for each version of the manuscript, as well as any author responses to peer review comments. Referees can decide whether or not they wish to be named on the peer review history document.

LANGUAGE EDITING AND SUPPORT FOR PUBLICATION: If you would like help with English language editing, or other article preparation support, Wiley Editing Services offers expert help, including English Language Editing, as well as translation, manuscript formatting, and figure formatting at www.wileyauthors.com/eeo/preparation. You can also find resources for Preparing Your Article for general guidance about writing and preparing your manuscript at www.wileyauthors.com/eeo/prepresources.

REVISION CHECKLIST: Upload a full Response to Referees file. To create your 'Response to Referees' copy all the reports, including any comments from the Senior and Reviewing Editors, into a Microsoft Word, or similar, file and respond to each point, using font or background colour to distinguish comments and responses and upload as the required file type.

Please upload two versions of your manuscript text: one with all relevant changes highlighted and one clean version with no changes tracked. The manuscript file should include all tables and figure legends, but each figure/graph should be uploaded as separate, high-resolution files.

You may also upload:

- 'Potential Cover Art' for consideration as the issue's cover image
- Appropriate Supporting Information (video, audio or data set: see https://jp.msubmit.net/cgi-bin/main.plex?form_type=display_requirements#supp).

We look forward to receiving your revised submission.

If you have any queries, please reply to this email and we will be pleased to advise.

Yours sincerely,

Bjorn Knollmann
Senior Editor
The Journal of Physiology

EDITOR COMMENTS

Reviewing Editor:

Thank you for this nice perspectives piece. It is a valuable addition to the paper. Please address the minor issues identified by the reviewer. Thanks.

Senior Editor:

I concur with the reviewing editor.

REFEREE COMMENTS

Referee #1:

The authors summarize and highlight key points from Nilsson et al. (2025), adding valuable clinical insights and additional autonomic-focused background. The review provides depth while clearly presenting the essential findings relevant to clinical practice. A few concerns are noted below.

• Line 26: Three senior authors are listed on Nilsson et al., including Dr. David Mendelowitz. Please revise to, "Recent seminal work by the Schunke, Kay and Mendelowitz' team...."

• Line 37: To clarify, the CVNs in the DMNX are activated by PVN neurons that co-release oxytocin and glutamate which excite oxytocin receptors on the CVNs. Nilsson et al (2025) do not show that CVNs are activated by 'activating oxytocin receptors in PVN fibers.'

• Line 53: This sentence is misleading - the first part summarizes HR indices altered by prediabetes and attenuated by Treatment. The second part describes data unaffected by prediabetes, so it wouldn't be expected to be attenuated by Treatment.

(a) Correct - HR and HRV were altered due to prediabetes, and were restored by Treatment to levels found in control animals.

(b) "...while blood pressure and exercise tolerance remained unaffected." -To clarify, blood pressure and exercise tolerance were unchanged by the development of prediabetes, so it is not expected that Treatment would have an effect.

• Line 77: It should be noted that this team has also published research on the intranasal delivery of oxytocin for patients with obstructive sleep apnea1,2.

1. Jain V, Kimbro S, Kowalik G, Milojevic I, Maritza Dowling N, Hunley AL, Hauser K, Andrade DC, Del Rio R, Kay MW, Mendelowitz D. Intranasal oxytocin increases respiratory rate and reduces obstructive event duration and oxygen desaturation in obstructive sleep apnea patients: a randomized double blinded placebo controlled study. Sleep Med. 2020 Oct;74:242-247. PMID: 32862007

2. Jain V, Marbach J, Kimbro S, Andrade DC, Jain A, Capozzi E, Mele K, Del Rio R, Kay MW, Mendelowitz D. Benefits of oxytocin administration in obstructive sleep apnea. American Journal of Physiology-Lung Cellular and Molecular Physiology. American Physiological Society; 2017 Nov;313(5):L825-L833.

END OF COMMENTS

The authors summarize and highlight key points from Nilsson et al. (2025), adding valuable clinical insights and additional autonomic-focused background. The review provides depth while clearly presenting the essential findings relevant to clinical practice. A few concerns are noted below.

- Line 26: Three senior authors are listed on Nilsson et al., including Dr. David Mendelowitz. Please revise to, "Recent seminal work by the Schunke, Kay and Mendelowitz' team…."
- Line 37: To clarify, the CVNs in the DMNX are activated by PVN neurons that co-release oxytocin and glutamate which excite oxytocin receptors on the CVNs. Nilsson et al (2025) do not show that CVNs are activated by 'activating oxytocin receptors in PVN fibers.'
- Line 53: This sentence is misleading – the first part summarizes HR indices altered by prediabetes and attenuated by Treatment. The second part describes data unaffected by prediabetes, so it wouldn't be expected to be attenuated by Treatment.

  (a) Correct - HR and HRV were altered due to prediabetes, and were restored by Treatment to levels found in control animals.

  (b) "…while blood pressure and exercise tolerance remained unaffected." -To clarify, blood pressure and exercise tolerance were unchanged by the development of prediabetes, so it is not expected that Treatment would have an effect.

- Line 77: It should be noted that this team has also published research on the intranasal delivery of oxytocin for patients with obstructive sleep apnea[1,2].

1. Jain V, Kimbro S, Kowalik G, Milojevic I, Maritza Dowling N, Hunley AL, Hauser K, Andrade DC, Del Rio R, Kay MW, Mendelowitz D. Intranasal oxytocin increases respiratory rate and reduces obstructive event duration and oxygen desaturation in obstructive sleep apnea patients: a randomized double blinded placebo controlled study. Sleep Med. 2020 Oct;74:242–247. PMID: 32862007

2. Jain V, Marbach J, Kimbro S, Andrade DC, Jain A, Capozzi E, Mele K, Del Rio R, Kay MW, Mendelowitz D. Benefits of oxytocin administration in obstructive sleep apnea. American Journal of Physiology-Lung Cellular and Molecular Physiology. American Physiological Society; 2017 Nov;313(5):L825–L833.

Response to Referees

EDITOR COMMENTS

Reviewing Editor:

Thank you for this nice perspectives piece. It is a valuable addition to the paper. Please address the minor issues identified by the reviewer. Thanks.

Senior Editor:

I concur with the reviewing editor.

Response: Thank you for the opportunity to complete this perspectives piece. We appreciate the support and will look to address the minor issues noted here.

REFEREE COMMENTS

Referee #1:

The authors summarize and highlight key points from Nilsson et al. (2025), adding valuable clinical insights and additional autonomic-focused background. The review provides depth while clearly presenting the essential findings relevant to clinical practice. A few concerns are noted below.

• Line 26: Three senior authors are listed on Nilsson et al., including Dr. David Mendelowitz. Please revise to, "Recent seminal work by the Schunke, Kay and Mendelowitz' team...."

Response: Will change sentence to: “Recent seminal work by the Schunke, Kay and Mendelowitz’ team demonstrated that targeting paraventricular nucleus (PVN) oxytocinergic neurons through chemogenic induction to manipulate cardiac cholinergic activation confers cardioprotective benefits in myocardial infarction (MI), hypertensive, and obstructive sleep apnea (OSA) models.”

• Line 37: To clarify, the CVNs in the DMNX are activated by PVN neurons that co-release oxytocin and glutamate which excite oxytocin receptors on the CVNs. Nilsson et al (2025) do not show that CVNs are activated by 'activating oxytocin receptors in PVN fibers.'

Response: Will change sentence to: “In rats, the CVNs in the DMNX are activated by PVN neurons that co-release oxytocin and glutamate which, in turn, excite oxytocin receptors on the CVNs, allowing PVN input to bypass sympathetic pathways and predominately stimulate parasympathetic tone.”

• Line 53: This sentence is misleading - the first part summarizes HR indices altered by prediabetes and attenuated by Treatment. The second part describes data unaffected by prediabetes, so it wouldn't be expected to be attenuated by Treatment.

(a) Correct - HR and HRV were altered due to prediabetes, and were restored by Treatment to levels found in control animals.

(b) "...while blood pressure and exercise tolerance remained unaffected." -To clarify, blood pressure and exercise tolerance were unchanged by the development of prediabetes, so it is not expected that Treatment would have an effect.

Response: Will change sentence to: “The assessment of cardiac parasympathetic tone revealed that heart rate variability and resting heart rate, altered in the prediabetic condition, were restored to control levels by treatment. In contrast, blood pressure and exercise tolerance were unaffected by the development of prediabetes and therefore remained unchanged following treatment.”

• Line 77: It should be noted that this team has also published research on the intranasal delivery of oxytocin for patients with obstructive sleep apnea[1,2].

1. Jain V, Kimbro S, Kowalik G, Milojevic I, Maritza Dowling N, Hunley AL, Hauser K, Andrade DC, Del Rio R, Kay MW, Mendelowitz D. Intranasal oxytocin increases respiratory rate and reduces obstructive event duration and oxygen desaturation in obstructive sleep apnea patients: a randomized double blinded placebo controlled study. Sleep Med. 2020 Oct;74:242-247. PMID: 32862007

2. Jain V, Marbach J, Kimbro S, Andrade DC, Jain A, Capozzi E, Mele K, Del Rio R, Kay MW, Mendelowitz D. Benefits of oxytocin administration in obstructive sleep apnea. American Journal of Physiology-Lung Cellular and Molecular Physiology. American Physiological Society; 2017 Nov;313(5):L825-L833.

Response: We would like to cite the additional papers by Jain et al. but understand that this would exceed the (5) references limit from the article's original instructions. If this is acceptable, we would be glad to reference this most recent work.

Will change ending paragraph to: "Future research directions may be tailored to simulating the effects shown here by activation of the PVN OXT neuronal network with drug delivery through alternative methods. This group previously explored intranasal delivery of oxytocin in obstructive sleep apnea (OSA) patients where it was found to decrease the severity and duration of obstructive events (Jain et al., 2020). Recent clinical trials have also studied its use for weight loss (McCormack et al., 2023). While this trial produced mixed results, the medication was well- tolerated in individuals and could provide a promising avenue for building strategies that target cardiac autonomic dysfunction in metabolic disorders.**"**

Dear Dr Wang,

Re: JP-TP-2025-289973R1 "**Heartfelt Intervention: A Role for PVN Oxytocin Neurons in the Prediabetic Heart**" by Grace K. Nielsen, Reesa M. Wilcox, and Han-Jun Wang

We are pleased to tell you that your paper has been accepted for publication in The Journal of Physiology.

Please see below for a minor amendment that can be made at proof stage.

TRANSPARENT PEER REVIEW POLICY: To improve the transparency of its peer review process, The Journal of Physiology publishes online as supporting information the peer review history of all articles accepted for publication. Readers will have access to decision letters, including Editors' comments and referee reports, for each version of the manuscript, as well as any author responses to peer review comments. Referees can decide whether or not they wish to be named on the peer review history document.

The last Word (or similar) version of the manuscript provided will be used by the Production Editor to prepare your proof. When this is ready you will receive an email containing a link to Wiley's Online Proofing System. The proof should be thoroughly checked and corrected as promptly as possible.

Authors should note that it is too late at this point to offer corrections prior to proofing. Major corrections at proof stage, such as changes to figures, will be referred to the Editors for approval before they can be incorporated. Only minor changes, such as to style and consistency, should be made at proof stage. Changes that need to be made after proof stage will usually require a formal correction notice.

All queries at proof stage should be sent to: TJP@wiley.com.

Are you on Twitter? Once your paper is online, why not share your achievement with your followers? Please tag The Journal (@jphysiol) in any tweets and we will share your accepted paper with our 30,000 followers!

If you would like to receive our 'Research Roundup', a monthly newsletter highlighting the cutting-edge research published in The Physiological Society's family of journals (The Journal of Physiology, Experimental Physiology and Physiological Reports), please click this link, fill in your name and email address and select 'Research Roundup': https://www.physoc.org/journals-and-media/membernews/

Yours sincerely,

Bjorn Knollmann
Senior Editor
The Journal of Physiology

EDITOR COMMENTS

Reviewing Editor:

Thank you for making these changes. We cannot exceed 5 references for this type of article.

Senior Editor:

The revision is acceptable. Thank you for your excellent contribution. Please limit references to 5 as per Journal guidelines. This can be done at the proof stage.

P.S. - You can help your research get the attention it deserves! Check out Wiley's free Promotion Guide for best-practice recommendations for promoting your work at www.wileyauthors.com/eeo/guide. You can learn more about Wiley Editing Services which offers professional video, design, and writing services to create shareable video abstracts, infographics, conference posters, lay summaries, and research news stories for your research at www.wileyauthors.com/eeo/promotion.

IMPORTANT NOTICE ABOUT OPEN ACCESS: To assist authors whose funding agencies mandate public access to published research findings sooner than 12 months after publication, The Journal of Physiology allows authors to pay an Open Access (OA) fee to have their papers made freely available immediately on publication.

The Corresponding Author will receive an email from Wiley with details on how to register or log-in to Wiley Authors Services where you will be able to place an order.

You can check if your funder or institution has a Wiley Open Access Account here: https://authorservices.wiley.com/author-resources/Journal-Authors/licensing-and-open-access/open-access/author-compliance-tool.html.
